# Deregulation of Negative Controls on TGF-β1 Signaling in Tumor Progression

**DOI:** 10.3390/cancers10060159

**Published:** 2018-05-25

**Authors:** Jiaqi Tang, Cody C. Gifford, Rohan Samarakoon, Paul J. Higgins

**Affiliations:** Department of Regenerative and Cancer Cell Biology, Albany Medical Center, 47 New Scotland Avenue, Albany, NY 12208, USA; tangj2@amc.edu (J.T.); gifforc@amc.edu (C.C.G.)

**Keywords:** transforming growth factor-β1, PTEN, PPM1A, Klotho, SMAD7, BMP7, Ski/Sno, BAMBI, tumor progression, cellular plasticity

## Abstract

The multi-functional cytokine transforming growth factor-β1 (TGF-β1) has growth inhibitory and anti-inflammatory roles during homeostasis and the early stages of cancer. Aberrant TGF-β activation in the late-stages of tumorigenesis, however, promotes development of aggressive growth characteristics and metastatic spread. Given the critical importance of this growth factor in fibrotic and neoplastic disorders, the TGF-β1 network is subject to extensive, multi-level negative controls that impact receptor function, mothers against decapentaplegic homolog 2/3 (SMAD2/3) activation, intracellular signal bifurcation into canonical and non-canonical pathways and target gene promotor engagement. Such negative regulators include phosphatase and tensin homologue (PTEN), protein phosphatase magnesium 1A (PPM1A), Klotho, bone morphogenic protein 7 (BMP7), SMAD7, Sloan-Kettering Institute proto-oncogene/ Ski related novel gene (Ski/SnoN), and bone morphogenetic protein and activin membrane-bound Inhibitor (BAMBI). The progression of certain cancers is accompanied by loss of expression, overexpression, mislocalization, mutation or deletion of several endogenous repressors of the TGF-β1 cascade, further modulating signal duration/intensity and phenotypic reprogramming. This review addresses how their aberrant regulation contributes to cellular plasticity, tumor progression/metastasis and reversal of cell cycle arrest and discusses the unexplored therapeutic value of restoring the expression and/or function of these factors as a novel approach to cancer treatment.

## 1. Introduction to TGF-β1 Signaling

Transforming growth factor β1 (TGF-β) is a member of a family of pleiotropic ligands that signal (as dimers) through serine/threonine kinase receptors to form heteromeric complexes composed of two ligand binding receptors (TGF-βRII) and two recruited receptors (TGF-βRI). Upon ligand engagement, TGF-βRII transphosphorylates and activates the kinase activity of TGF-βRI resulting in phosphorylation and mobilization of the transcriptional effectors mothers against decapentaplegic homolog (SMAD) proteins [1]. SMADs partition into three classes: (a) SMADs that directly interact with TGF-βRI and are subsequently activated by C-terminal phosphorylation to initiate gene expression are named receptor-regulated SMADs (R-SMADs) which include SMAD1, 2, 3, 5 and 8; (b) SMAD4, which participates in signaling by several TGF-β family members as a cofactor, facilitates nuclear translocation of phosphorylated R-SMADs and finally (c) the antagonistic SMADs (i.e., SMAD6 and 7) that negatively regulate the R-SMAD pathways to inhibit TGF-β signaling [2].

TGF-βRI phosphorylates SMAD2/3 at two C-terminal serines which is required to mediate association of SMAD2/3 with SMAD4 in mammalian cells [3,4]. SMAD2/3/4 complexes then translocate to the nucleus to act as sequence-specific DNA-binding proteins factors, facilitating the transcription of TGF-β family member responsive genes [5]. Overexpression of SMAD3 or SMAD4 alone, however, does not result in TGF-β1 target promoter activation, suggesting the requirement for additional co-factors. These include p300, CREB-binding protein (CBP), and P300/CBP-associated factor (P/CAF) that acetylate SMAD2 and SMAD3 at Lysine 19, inducing a conformational change in the DNA-binding MAD homology 1 (MH1) domain to promote interactions with their recognition motifs [6]. SMAD2/3/4, however, possess weak DNA-binding ability necessitating the participation of non-canonical (i.e., non-SMAD) transcriptional factors including Activator Protein 1 (AP-1), c-fos, c-Jun, p53, Yes-associated protein-Transcriptional coactivator with PDZ-binding motif (YAP-TAZ), TEA domain family (TEAD) and β-catenin to maintain effective controls on gene expression [7,8,9,10,11].

**Figure 1:**
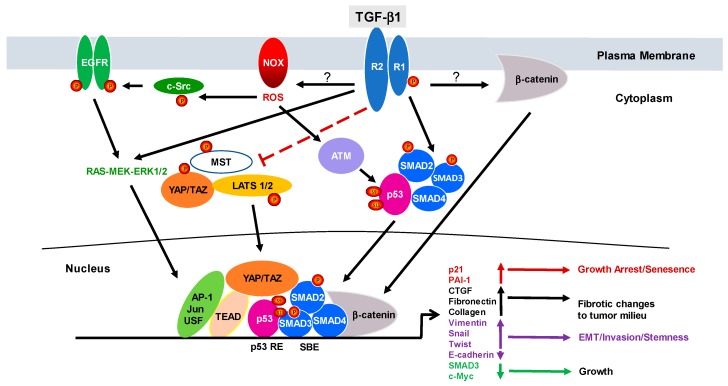
SMAD and non-SMAD Signaling Cooperation Downstream of TGF-β1 in Orchestrating Distinct Biological Responses. TGF-β1 ligand-receptor engagement initiates signaling via both canonical SMAD2/3 and non-canonical (e.g., NOX, p53, YAP/TAZ, EGFR, c-Src, MAPK) pathways impacting transcriptional activation or repression of number of target genes as indicated. Resulting biological outputs may include growth inhibition, oncogenic fibrotic reprogramming, epithelial plasticity and cell growth depending on the contextual role of TGF-β1 in carcinogenesis.

Non-canonical cascades are critically important for the initiation and progression of various TGF-β1-driven pathologies [7,8,9]. Generation of free radicals by NADP(H)oxidases downstream of TGF-β1 stimulation activates c-Src and Ataxia-telangiectasia mutated (ATM) kinases critical for signal propagation [8,11,12,13,14]. ATM directed p53 phosphorylation and subsequent assembly of p-p53 and pSMAD2/3 transcriptional complexes are essential to drive gene expression via SMAD and non-SMAD cooperation [12,13]. TGF-β1-mediated reactive oxygen species (ROS) generation also phosphorylates c-Src at Y418, which in turn transactivates Src kinase target epidermal growth factor receptor (EGFR) residues activating the MEK-ERK1/2 cascade [8,11,14]. AP-1, c-Jun and upstream stimulatory factor (USF) are known substrates of the ERK/JNK/p38 pathway. SMAD3 and 4 cooperate physically and functionally with c-Jun/c-Fos at the AP1-binding site to initiate TGF-β1-dependent transcription [15]. The Hippo pathway nuclear transducers YAP/TAZ, furthermore, are activated in response to TGF-β1 likely via inhibition of the core Hippo components (including the mammalian sterile 20-like/large tumor suppressor (MST/LATS) kinases) resulting in nuclear accumulation of YAP/TAZ. Since YAP/TAZ do not engage DNA directly, they form complexes with TEAD or SMAD2/3 s to dictate TGF-β1 target gene transcription (Figure 1) [16,17,18,19,20,21].

## 2. The Duality of TGF-β1 Signaling: From Cancer Suppressor to Tumor Promotor

Several excellent reviews have detailed the molecular basis for transition of TGF-β1 from an anti- to pro-tumorigenic effector [22,23,24,25]. As this information is readily available, the summary below highlights the key aspects of this phenotypic reprogramming in the context of its potential impact on the negative regulators of the TGF-β1 pathway.

Among the most prominent actions of the TGF-β1 signaling system is the pronounced effect on the cellular proliferative program. Under normal circumstances, TGF-β1 functions as a tumor suppressor via three basic mechanisms involving initiation of cell cycle arrest (primarily in G_1_), promotion of apoptosis and inhibition of cell immortalization [22,26,27].

G_1_ arrest results from the induction of the cyclin-dependent kinase inhibitors p15, p21 and p27 and inhibition of expression of the growth-promoting factors c-Myc and the inhibitor of DNA binding (ID) family of helix-loop-helix transcription proteins [23]. c-Myc repression may play a multifunctional role in the cytostatic program. c-Myc down-regulates p15 and p21 expression at the promoter level suggesting that TGF-β1-induced growth restriction may reflect up-regulation of p15 and p21 expression directly as part of the TGF-β1 response as well as the release of p15 and p21 from c-Myc suppression as a result of attenuation of c-Myc levels upon TGF-β1-stimulation [22]. TGF-β1 controls on the anti-apoptotic and anti-immortalization programs are similarly complex and both involve participation of members of the E2F family of transcription factors and signaling through the stress-activated protein kinase/c-Jun N-terminal kinase pathway [22,28]. This delicate tumor-suppressing homeostatic balance is upset in a wide spectrum of human cancers due to mutations, deletions, epigenetic alterations or expression variability in key elements including TGF-βRI and II, SMAD 2, 3 and 4 as well as TGF-β1 itself resulting in the transition of TGF-β1 from an anti- to pro-tumorigenic cytokine [22,28,29,30].

The pro-cancer progression consequences of aberrant TGF-β1 pathway signaling impacts virtually all cell types in the tumor microenvironment and involves redirected stromal remodeling, promotion of tumor cell plasticity (facilitating migration, invasion and metastasis), stimulation of angiogenesis, induced immunosuppression with loss of immunosurveillance and resistance to the TGF-β1-driven apoptotic and cytostatic programs [31]. In addition to core elements in the canonical TGF-β1 network, it is now evident that several negative regulators of TGF-β1 signaling are altered during tumorigenesis with subsequent consequences on tumor behavior and patient outcomes.

## 3. Relevance of Negative Regulators of TGF-β Signaling to Cancer Progression

**Figure 2:**
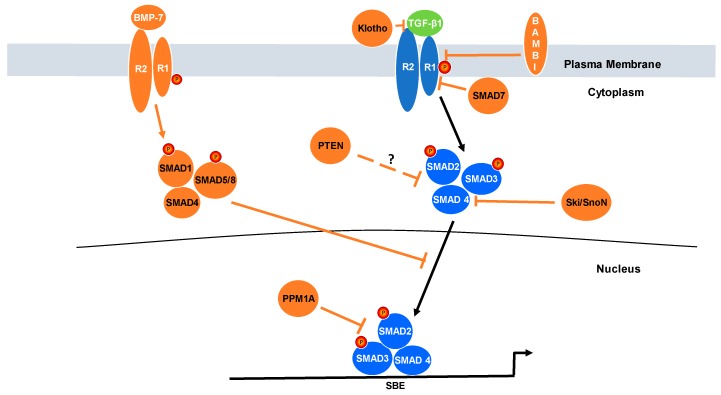
A Complex Network of Negative Regulators Fine-tunes TGF-β1-Driven Phenotypic Modifications in Cancer. TGF-β1-SMAD2/3 signal transduction (highlighted in blue) is subject to modification by a number of repressors (highlighted in orange) that function at the levels of the receptor or SMAD2/3 activation, nuclear translocation and promoter binding as depicted above. Deregulation of such negative controls could profoundly influence TGF-β1-induced genetic programs in tumorigenesis.

Earlier seminal findings identified several endogenous regulators that exert repressive activity on the TGF-β1 pathway at the levels of receptor activation (e.g., Smurf, Klotho, and SMAD7), SMAD2/3 phosphorylation (e.g., Ski/SnoN, BMP6/7, PPM1A, and PTEN) and nuclear translocation as well as pSMAD2/3 engagement (e.g., PPM1A) to target gene promoters in a manner that either stimulates or inhibits transcriptional outputs [32]. Such complex controls on the TGF-β1 network appear necessary to appropriately titrate the initiation, amplitude and duration of TGF-β1 pathway involvement in the tumor suppressor vs. pro-tumorigenic genomic programs. This review focuses on a discussion of seven such negative TGF-β1 signaling regulators and their impact on cellular phenotypic transitions and clinical outcomes (Figure 2).

## 4. Reciprocal Relationship between PTEN and TGF-β Signaling

Tumor suppressor phosphatase and tensin homologue (PTEN) on chromosome 10 is one of the most frequently mutated and/or deleted genes in human tumors [33]. PTEN represses the phosphoinositide 3-kinase (PI3K)-AKT pathway, by dephosphorylating phosphatidylinositol-3,4,5-triphosphate (PIP3) to phosphatidylinositol-4,5-triphosphate (PIP2) suppressing, thereby, AKT activation [34]. PTEN deficiency leads to hyperactivation of AKT, impacting critical cellular processes including proliferation, growth, survival, migration, apoptosis and cell cycle transit [35,36].

Recent findings established a bi-directional pathophysiologic link between the PTEN and TGF-β1 pathways in cancer progression. Increased serum TGF-β1 levels are consistently and strongly associated with poor prognosis in prostate cancer patients [37]. Proteomic analysis of mouse prostate carcinomas from prostate-specific PTEN-deleted mice revealed upregulation of SMAD2/3, SMAD4, pSMAD2/3 compared to tumors from control animals [38]. Transgenic mice with a conditional prostatic deletion of both SMAD4 and PTEN developed more aggressive and metastatic adenocarcinomas compared to PTEN deletion alone [38]. Prostate-specific loss of PTEN results in AKT activation [39] and constitutive AKT1 signaling in the mouse prostatic epithelium leads to mobilization of the TGF-β1 pathway as evident by an increase in TGF-β receptor II (TGF-βRII) and SMAD4 expression [40]. These findings, however, are not exclusive to cancer as renal epithelial tubular PTEN deficiency in various kidney injury models promoted a fibrotic phenotype and epithelial growth arrest (dysfunction) via AKT, SMAD3- and p53-dependent mechanisms [41]. 

Although inactivation of TGF-βRII and PTEN loss of expression are common in human colorectal cancer, ablation of TGF-βRII or PTEN alone in the mouse intestinal epithelium has little to no effect on tumorigenesis [42]. Intestinal epithelial-specific inactivation of TGF-βRII in the context of PTEN loss leads to adenocarcinoma development in mice, suggestive of functional cooperation between PTEN and TGF-β1 deregulation in cancer progression. Tumors from double-transgenic mice with TGF-βRII and PTEN intestinal-specific deletion also exhibited decreased apoptosis (as highlighted by a decrease in cleaved caspase-3) and downregulation of expression of the cyclin-dependent kinase inhibitors (e.g., p15^INK4B^, p21^CIP1^ and p27^KIP1^) compared to control animals [42].

TGF-β1 overexpression is a frequent molecular hallmark in pancreatic malignancies [43]. In pancreatic adenocarcinomas, there is a reduction of PTEN mRNA compared to normal pancreas from organ donors [44] likely orchestrated by TGF-β1 via PKC-α activation [45]. Furthermore, PTEN mRNA is also attenuated in the pancreas of transgenic mice that overexpress TGF-β1 in pancreatic B cells relative to control mice, confirming that TGF-β1 downregulates PTEN both in vitro and in vivo [44].

In type II endometrial cancer patients, increased plasma TGF-β1 expression is associated with advanced-stage disease [46]. TGF-β1 stimulation promotes a migratory phenotype in endometrial tumor cells while downregulating both PTEN mRNA and protein expression [47]. Disruption of the canonical TGF-β1→SMAD2/3/4 axis attenuated TGF-β1-mediated PTEN down-regulation as did inhibition of TGF-β1-MEK-ERK1/2 signaling in type II endometrial cancer cells [47]. Ectopic overexpression of PTEN, moreover, abolishes TGF-β1-induced motility in vitro, suggesting that PTEN is a negative regulator of the TGF-β1-stimulated pro-migration pathway. Similarly, PTEN also suppresses TGF-β1 signaling in kidney fibrosis [41].

**Figure 3:**
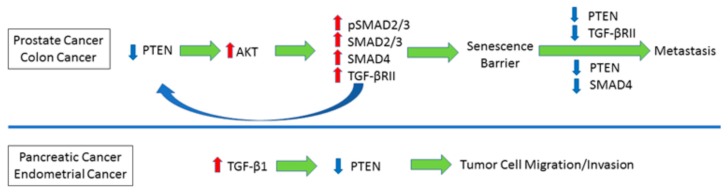
Extensive Cross-Talk between PTEN and TGF-β1 in Cancer Progression. PTEN silencing, as evident during prostate cancer, promotes SMAD3, SMAD2/3 or TGF-βRII expression, establishing cancer cell senescence. Concurrent loss of either PTEN and SMAD4 or PTEN and TGF-βRII breaches this barrier against tumor progression and promoted metastasis. TGF-β1 hyperactivation, as observed in pancreatic cancer, could downregulate PTEN via transcriptional or non-transcriptional (e.g., protein degradation) mechanisms, facilitating cancer cell invasion.

In summary, in both prostate and colon cancer, PTEN loss is causatively linked to increased TGF-β1 signaling (e.g., TGF-βRII, SMAD2/3, SMAD4 upregulation). SMAD4 or TGF-βRII deficiency in the context of PTEN loss in the prostate tissue leads to escape of the senescence barrier imposed by the PTEN loss, promoting metastasis. TGF-β1, frequently upregulated in pancreatic and endometrial cancer, also attenuated PTEN expression via different mechanisms, influencing, thereby, cellular migratory/invasive traits (Figure 3).

## 5. Protein Phosphatase Magnesium-Dependent 1A (PPM1A)

PPM1A, first identified in rat liver and human teratocarcinoma libraries [48], is a serine threonine phosphatase, that regulates several signaling pathways including the AMP-activated protein kinase (AMPK), p38, JNK, and p53 networks [49]. PPM1A is also a C-terminal SMAD2/3 phosphatase that, thereby, inhibits TGF-β1 signal transduction and attenuates TGF-β1 target gene expression [50]. Recent studies implicate PPM1A as a major pathophysiologic contributor to TGF-β1 pathway activation in human malignancies. 

PPM1A deficiency is apparent in bladder cancer correlating with SMAD2/3 activation, muscular invasion and poor outcomes [51]. Similarly, experimental wound closure and trans-well migration assays confirmed that PPM1A knockdown enhanced TGF-β1-driven invasive properties [51]. Stable silencing of PPM1A in T4 and 5637 bladder cancer cells, in fact, promoted EMT/epithelial plasticity in response to TGF-β1 while increasing tumor growth and metastasis [51].

PPM1A expression is significantly downregulated in human hepatitis C virus (HCV)-related hepatocellular carcinomas (HCC) compared to normal liver tissues [52]. PPM1A depletion in HCC cells also induces an EMT-like phenotype, which is further exacerbated upon TGF-β1 stimulation [52]. While the underlying mechanism is unclear, it appears that the HCV nonstructural protein 3 (NS3) interacts with PPM1A to promote its ubiquitin-dependent proteasomal degradation.

In human prostate cancer, distant metastases exhibit significantly lower PPM1A expression levels compared to primary tumors [53]. Intracardiac delivery of PPM1A-expressing human prostate cancer cells into nude mice resulted in a reduced incidence of metastases relative to non-engineered tumor cells; the tumor apoptotic rate was also significantly higher compared to tumors that developed in animals injected with empty vector-transduced prostate cells [53]. These experiments suggest that restoration of PPM1A expression may have clinical utility as an anti-tumor strategy, at least in prostate cancer.

PPM1A deficiency is similarly associated with advancing tumor grade and poor prognosis in pancreatic ductal adenocarcinoma (PDAC) [54]. PPM1A expression is significantly decreased in PDAC distal metastases compared to the levels in matched primary tumors, suggesting that PDAC spread is associated with PPM1A down-regulation [54].

Collectively, it appears that PPM1A loss or reduced expression is common in several epithelial malignancies promoting migration, plasticity and metastases likely via aberrant activation, or retention, of TGF-β1 signaling. Rescue of PPM1A expression, conversely, attenuates TGF-β1 signaling and cancer cell invasion. PPM1A is a repressor of the TGF-β1 pathway is not exclusive to cancer. PPM1A expression is also lost in kidney fibrosis and PPM1A deregulation in renal epithelium leads to a dedifferentiated phenotype, SMAD3 activation, and subsequent potentiation of TGF-β1-driven fibrotic gene expression [55].

## 6. SMAD7

SMAD7 is an antagonist of the TGF-β1 signaling pathway forming stable complexes with TGF-βRI to inhibit R-SMAD phosphorylation and downstream signaling [56,57]. SMAD7 also binds/recruits the type E3 ubiquitin ligase ubiquitination regulatory factors (Smurfs) to degrade TGF-βRI via the proteasomal complex terminating, thereby, TGF-β1 signaling [58]. Defining the actual function of SMAD7 is complicated by its divergent activities in different tumor types; in melanoma and breast cancer, SMAD7 exhibits anti-tumor properties, whereas in skin, colon, pancreas, and endometrial malignancies, SMAD7 acts as a tumor promoter [59].

Autocrine TGF-β1 signaling via the SMAD pathway promotes melanoma progression and metastasis [60]. In human melanoma cells, constitutive SMAD2/3 signaling is mitigated by SMAD7 stable overexpression, resulting in decreased cell invasion and secretion of MMP-9/MMP-2 [61]. Furthermore, the tumor burden in nude mice subcutaneously injected with SMAD7-expressing human melanoma cells was reduced compared to animals implanted with control melanoma xenografts [61] while cardiac ventricular inoculation of SMAD7-expressing human melanoma cells exhibited attenuated bone metastasis relative to empty vector-transduced control cells [62].

TGF-β1 signaling is also hyperactivated in breast cancer, which drives cancer progression and metastasis [63]. Novel regulators of SMAD7, including OTU domain- containing protein 1 (OTUD1) and ubiquitin-specific protease 26 (USP26), attenuate TGF-β1-mediated aggressive phenotypes in breast cancer [64,65]. Expression of OTUD1, a metastasis suppressor, is lost in the later stages of breast cancer. OTUD1 is a deubiquitinase and among its targets is SMAD7, and OTUD1 deficiency destabilizes SMAD7 and potentiates TGF-β1-driven metastasis [64]. USP26 regulates SMAD7 stability, by deubiquitinating SMAD7, mitigating TGF-β1-induced migration and invasion in breast carcinoma cell lines [65].

In colon cancer, SMAD7 deletion predisposes to a favorable prognosis compared to patients with two copies of the gene, whereas patients with SMAD7 gene amplification exhibit significantly worse outcomes [66]. Indeed, genomic approaches identified specific SMAD7 alleles that impact colorectal cancer risk [67]. SMAD7 protein levels are markedly upregulated in human colorectal cancer relative to matched adjacent tissues similar to findings in colorectal cancer cell lines [68]. Indeed, subcutaneous delivery of SMAD7-expressing cells derived from non-tumorigenic human colon adenocarcinoma promoted tumor growth in nude mice, whereas control vector cells failed to form tumors [69]. SMAD7 reduces TGF-β1-mediated cell cycle arrest, via blockade of TGF-β1-induced suppression of CDK4, cyclin D1, and c-Myc, while also repressing p21^Cip1^ expression [69]. The pro-tumorigenic phenotype driven by SMAD7-overexpression is likely due to suppression of TGF-β1-mediated cell cycle arrest/growth inhibition. Moreover, splenic injection of SMAD7-overexpressing cells in nude mice leads to a significant increase in liver metastasis, compared to non-engineered parental and vector control-expressing cells [70]. Intraperitoneal injection of SMAD7 antisense oligonucleotides, in contrast, attenuates tumor burden in mice transplanted with human colorectal cells-derived xenografts, likely a reflection of, in part, a reduction in tumor cell growth [68].

Transgenic mice expressing a keratin K5 promoter-driven SMAD7 construct develop epidermal hyperplasia as well as delayed hair follicle formation [71] and downregulate TGF-βRI and II protein levels (but not mRNA transcript abundance) in the skin. SMAD7 overexpression, moreover, inhibits TGF-β1/SMAD signaling as evidenced by reduced p21 transcripts and increased c-myc expression likely resulting in SMAD7-driven epidermal hyperplasia.

Pancreatic-specific overexpression of SMAD7 in mice leads to inhibition of TGF-β1 signaling and emergence of foci of premalignant pancreatic intraepithelial neoplasia [72]. Immunohistochemical staining and proteomic analysis, moreover, confirmed that pSMAD2 is downregulated in the pancreas of SMAD7 transgenics corresponding with enhanced proliferation of ductal and acinar cells.

Collectively, it appears that tumor cell type dictates SMAD7 function as a tumor promoter or suppressor. In breast cancer, SMAD7 is destabilized by loss of expression of specific deubiquitinases, augmenting TGF-β1 signaling and the acquisition of metastatic behavior. In colon and pancreatic cancer, SMAD7 upregulation is associated with increased oncogenic potential. Although the precise mechanisms are not clear, repression of TGF-β1 signaling and growth inhibition mediated by SMAD7 is likely a key molecular feature in the development of malignancies involving the colon and pancreas.

## 7. Klotho

The Klotho gene encodes a single-pass β-glucuronidase-like transmembrane protein involved in calcium and phosphate metabolism [73]. The two extracellular homologous KL1 and KL2 domains (that have no known intracellular signaling capabilities) are proteolytically cleaved to function as endocrine factors [74]. Klotho was originally identified as a negative regulator of aging and Klotho deficient mice exhibit a short lifespan with a phenotype that resembles premature aging [75] while overexpression increases longevity and affords protection from many age-related diseases [75,76,77,78,79,80,81,82,83]. Indeed, Klotho deregulation (loss of expression) is a common event in breast [84], gastric [85] and colorectal malignancies [86]. 

Several groups reported a downregulation of Klotho in hepatocellular carcinoma (HCC) and Klotho deficiency in patients with HCC correlates with poor survival. Klotho silencing in HCC cells via siRNA enhanced tumor cell growth in vitro. Forced overexpression of Klotho decreased proliferation of hepatoma cells by preventing downstream Wnt signaling. Similar efficacy is evident in vivo. HCC tumor-bearing mice receiving intratumoral injections of soluble Klotho twice weekly for 4 weeks exhibited a significant reduction in tumor size compared to saline controls, demonstrating that Klotho is a tumor suppressor in hepatocellular carcinoma [87].

Evidence indicates that ligands from multiple growth factor-activated pathways (e.g., TGF-β, FGF, IGF and Wnt) directly bind to Klotho to modulate downstream signaling suggesting an intricate network of Klotho involvement [83]. Klotho preferentially binds multiple growth factor receptors on the cell surface, including FGFR1 [77], IGF [88], TGF-βRII [79] and Wnt [76,87], to inhibit signaling in normal tissues. Loss of Klotho, however, results in enhanced expression or activity of these growth factors, leading to disease progression.

Klotho also interacts with TGF-β1RII in renal epithelial cells, likely repressing TGF-β1 signaling (Figure 2) [79]. Loss of Klotho in HCC may well attenuate repressive effects on TGF-β receptors. Klotho rescue or ectopic expression also reduces migratory activity and anti-tumorigenic effects in lung cancer in vitro. A significantly impaired EMT or plastic response to TGF-β stimulation was evident in lung adenocarcinoma cells (A549) stably transfected with a Klotho expression vector. Cells co-stimulated with TGF-β and Klotho similarly exhibited diminished migration compared to controls without Klotho [79]. Tail vein injection of A549 Klotho-expressing cells into athymic mice resulted in significantly longer survival and decreased number of metastases compared to the empty vector control [79]. 

Loss of Klotho expression likely occurs through epigenetic silencing (via promoter methylation) [84,85,86]. It appears that TGF-β1 is a major regulator of Klotho silencing based recent studies in renal fibrotic disease where Klotho is lost in the tubular epithelium [79]. TGF-β1, in this context, directly represses miR-152 and miR-30 (two microRNAs that normally down-regulate the DNA methyltransferases DNMT1 and DNMT3a) resulting in increased methyltransferase activity and hypermethylation of the Klotho promoter [89]. This mechanism allows for stable global klotho silencing and an amplified, irreversible fibrotic response.

In summary, Klotho appears to be a tumor suppressor; loss of klotho, readily evident during cancer progression, promotes migration, invasion and tumor growth via activation of growth factor signaling pathways (e.g., Wnt and TGF-β1). Klotho rescue, conversely, blunts cancer progression. Since there is a correlation between enhanced TGF-β1 activity and Klotho silencing, it is attractive to speculate that TGF-β1 promotes loss of Klotho through promoter hypermethylation, alleviating Klotho’s repressive effects on growth factor signaling.

## 8. BMP7

TGF-β1 and BMP-7 (bone morphogenetic protein-7), two major members of the TGF-β family of receptor ligands, activate downstream signaling pathways and their respective target genes to counter-regulate biological outcomes [90]. Loss of this homeostatic control is a key feature in the development of several chronic diseases. BMPs form heteromeric complexes that initiate receptor kinase activity initiating downstream signaling [91]. TGF-β1 and BMP7 engage a complex genomic program, largely due to differential SMAD involvement (e.g., SMAD2/3 vs. SMAD1/5/8, respectively) [92]. BMP7-induced SMAD1/5/8 phosphorylation opposes TGF-β1-mediated phosphorylation of SMAD2/3 down-modulating, thereby, SMAD2/3-driven gene reprogramming (Figure 2).

BMPs function as tumor suppressors or oncogenes dependent on cell and tissue type, microenvironmental influences, tumor stage or epigenetic background, highlighting the complexity of the dual roles that various BMPs play in cancer progression [93,94]. BMP7 reduces tumor growth and down-regulates expression of progenitor cell biomarkers in subcutaneous and orthotopic glioblastoma xenografts. Indeed, exposure of glioblastoma stem-like cells to recombinant BMP7 in vitro increased SMAD1/5/8 phosphorylation, inhibited cell proliferation, stimulated glioblastoma differentiation, decreased neurosphere formation and attenuated expression of the stem-like genes Nanog, SOX2, Nestin and Olig2 [95]. Intracranial delivery of BMP7 to tumor xenografts generated by intracerebral injection of human glioblastoma stem-like cells significantly reduced growth, invasion and the overall mitotic index [95]. These data suggest that loss of BMP7 is a critical factor in glioblastomas progression and likely facilitates persistence of progenitor cell stemness.

BMP7 deficiency accelerates tumorigenesis and negatively correlates with patient survival in prostate cancer. Human prostatic carcinomas generally exhibit significantly lower levels of BMP7 transcripts compared to the normal-appearing gland; daily injections of recombinant BMP-7 protein reduced tumor burden and metastasis in mice transplanted with prostate cancer cell lines directly into the prostate or bone. While the mechanism is unclear, BMP7 increases E-cadherin expression and reduces vimentin levels in prostate tumor cells in vitro suggesting down-modulation of the plastic phenotype [96].

Consistent with these findings, loss of BMP7 in breast cancer appears to be responsible for EMT and bone metastases. In mice, delivery of MDA-231 cells via tail vein, mammary fat pad or intraosseous injection results in lesion formation and significant secondary metastatic growth. Implantation of MDA-231 cells engineered to overexpress BMP7, or injection of recombinant BMP7, however, reduced (by 50%) the number of osteolytic lesions, overall tumor burden and the total osteolytic area [97]. In vitro modeling confirmed that metastatic potential positively correlates with vimentin expression and loss of E-cadherin; addition of TGF-β1 enhanced these aggressive phenotypic characteristics [97]. Importantly, BMP7 inhibits the TGF-β1-mediated expression of the EMT genes ZEB1/2, Snail1/2, N-cadherin, vimentin, and collagen I while restoring E-cadherin levels and inhibiting growth and invasion [96,97,98], demonstrating that BMP7 plays a critical role in inhibiting metastasis and prevents the expression of plasticity-related genes both in vitro and in vivo.

EMT in breast cancer cells is associated with the expression of microRNA-137 which inhibits BMP7 expression resulting in enhanced invasion. Inhibition of miRNA-137 rescued BMP7 expression and repressed tumor cell migration [98]. siRNA-mediated TGF-β1 knockdown significantly reduced miRNA-137 expression implicating the TGF-β1 pathway in miRNA-137 control [99]. Downregulation of BMP7 in breast cancer may be attributed to the increased expression of miRNA-137, which is mediated by TGF-β1 signaling.

Loss of BMP7 in glioblastoma, breast, and prostate cancers, therefore, predisposes to enhanced EMT and acquisition of invasive/metastatic traits suggesting a protective role for BMP7 in cancer progression. The opposing cellular responses to TGF-β1 and BMP7 suggest that clarification of the BMP7 signaling pathway may identify novel therapeutic approaches for the treatment of both fibroproliferative disorders and malignant disease.

## 9. Ski/SnoN

Ski (Sloan-Kettering Institute proto-oncogene) and the closely related SnoN (Ski related novel gene) share a 50% homology; both are potent negative regulators of TGF-β signaling [100,101]. Members of the Ski family suppress SMAD function by binding to the N-terminus of R-Smads (e.g., Smad2/3) and the SAND-like domain of SMAD4 to block complex formation and transcriptional responses [102]. Ski and SnoN are upregulated by TGF-β signaling where they act in a negative feedback manner. TGF-β, however, also downregulates Ski and SnoN via the ubiquitin-proteasomal pathway which may provide for persistent signaling [101,103,104]. Fine control of these two regulators can impact the effects of TGF-β on cellular growth programs. As a result, Ski and SnoN can be both oncogenic as well as tumor suppressive and their expression is elevated in several cancers [100].

Inhibition of Ski in pancreatic cancer inhibits tumor growth. Increased Ski expression in human pancreatic cancer, for example, correlates with poor survival while transfection of pancreatic tumor cells with Ski siRNA augments TGF-β1 signaling and promoted TGF-β1-mediated p21 expression and growth inhibition [105]. Transplantation of Ski knockdown pancreatic cancer cells into mice, moreover, significantly reduced the subsequent tumor burden and increased the necrotic area [105].

In Barrett’s esophagus, Ski and SnoN are markedly elevated in low grade dysplasia but decreased or absent throughout the metaplasia-dysplasia-adenocarcinoma sequence, suggesting that a stage-dependent expression pattern may be a causative factor in the progression of Barrett’s esophagus [106]. This leads to the speculation that repression of the TGF-β1-induced anti-proliferative response may be mechanistically linked to the progression of this disease in the context of Ski/SnoN up-regulation. The tumor suppressive effects of Ski/SnoN are evident, moreover, in heterologous Ski and SnoN mice which are more susceptible to carcinogen-induced tumorigenesis than wild type controls [107,108].

## 10. Bone Morphogenetic Protein and Activin Membrane-Bound Inhibitor (BAMBI) and Rac1b

BAMBI is a transmembrane TGF-β pseudoreceptor homologous in structure to TGF-β-family type I receptors but lacking an intracellular kinase domain [109]. BAMBI antagonizes TGF-β1 signaling by interacting with TGF-β receptors preventing receptor complex formation, thereby, inhibiting downstream signaling [109]. The function of BAMBI in cancer progression, similar to SMAD7 and BMP7, appears to be context dependent; in colorectal malignancies BAMBI is pro-tumorigenic whereas in the lung and bladder cancer BAMBI exhibits anti-tumor properties.

BAMBI levels are significantly elevated in human colorectal tumors compared to the corresponding non-malignant tissue [110] where it correlates with local invasiveness and poor prognosis [111]. In human colorectal cell lines, inhibition of β-catenin signaling represses BAMBI expression, suggesting a link between the β-catenin pathway and TGF-β1 signaling [110]. Overexpression of BAMBI in human colorectal carcinoma cells increases migration in vitro and metastatic incidence in nude mice [112]. This relationship appears causative as siRNA-mediated BAMBI knockdown in colon carcinoma cells decreases both proliferation (as evidenced by reduced Ki67 and PCNA expression) and cellular motility while increasing caspase 3,8,9-initiated apoptosis [113]. TGF-β signaling is upregulated upon BAMBI inhibition resulting in elevated levels of pSMAD2/3 and E2F4/5 and c-MYC suppression [113]. Moreover, subcutaneous injection of BAMBI siRNA pre-treated colon carcinoma cells in nude mice dramatically reduced tumor growth and metastases [113].

In sharp contrast to colorectal cancer, human lung tumors have reduced BAMBI expression. Immunohistochemical analysis revealed an almost complete absence of BAMBI protein in human NSCLC (non-small cell lung cancer) tissue compared to non-cancerous controls, corresponding with decreased mRNA levels, increased promoter hypermethylation and upregulation of the EMT genes N-cadherin, TWIST, MMP-9, osteopontin, and SOX4 [114]. Upregulation of TGF-β1 and SMAD2/3/4, increased SMAD2/3 phosphorylation and a loss of BAMBI expression were prominent in the lung cancer cell lines A549 and H1975. In BAMBI knockdown cells, TGF-β1 hyper-induced SMAD2/3 activation and EMT related gene expression and stimulated migration. Stable overexpression of GFP-BAMBI in these cells, in contrast, resulted in a significant decrease in SMAD activation and EMT gene transcription in response to TGF-β1 and reduced migration speed [114]. Nude mice injected with GFP-BAMBI expressing cells formed fewer nodules and had a reduced tumor burden in the lungs compared to GFP controls suggesting that BAMBI is a potent inhibitor of TGF-β1-mediated EMT and invasion in vitro and in vivo [114].

Bladder tumors display a BAMBI expression pattern similar to that seen in lung cancer. Immunohistochemical analysis revealed a stage-dependent expression pattern, where BAMBI is present in low grade tumors but absent in high grade, invasive malignancies. As was the case in lung carcinomas, transient transfection of BAMBI siRNA markedly reduced the migratory response to TGF-β1 in bladder cancer cell lines [115].

These studies collectively suggest that elevated BAMBI expression downregulates TGF-β1 signaling and promotes tumor progression in colorectal cancer cells while, in the lung and bladder, loss of BAMBI promotes tumorigenesis by TGF-β1 hyperactivation, induction of EMT and acquisition of pro-invasive properties.

Rac1b, an active and alternatively spliced isoform of Rac1-GTPase, appears to differentially impact cancer properties depending on the tumor type. In pancreatic ductal adenocarcinoma, Rac1b upregulation represses TGF-β1-stimulated EMT and motility through inhibition of both canonical (SMAD) and non-canonical (MAPK) signaling [116,117]. In contrast, MMP-3-induced Rac 1b expression promotes lung adenocarcinoma; the upregulation of Rac1b decreased the expression of E-cadherin while promoting the transition to a mesenchymal phenotype, increased migration, and induced transcription of EMT-related genes [118,119]. Thus, further definition of the molecular basis of tissue-specific Rac1b regulation may provide context-dependent therapeutic opportunities to attenuate TGF-β1-mediated pro-tumorigenic properties. 

## 11. Pathological Significance, Perspectives and Conclusions

TGF-β1 exerts both protective and detrimental effects during cancer growth. The anti-tumorigenic actions of TGF-β1 reflect upregulation of the growth arrest gene p21 as well as the reduced expression of proliferative factors such as c-myc and suppression of inflammation. TGF-β1-induced pro-tumorigenic functions, in contrast, are linked to induced epithelial plasticity and the acquisition of cellular migration/metastatic properties coupled with fibrotic changes to the tumor microenvironment. These findings not only question the feasibility of global targeting of the TGF-β1 pathway as a plausible anti-cancer strategy but underscore the need to identify malignancy-specific approaches to target the pro-tumorigenic effects TGF-β1 signaling while retaining anti-inflammatory and anti-proliferative properties. Definition of the molecular basis of inhibitory controls on the TGF-β1 network provides unique opportunities to fine tune cytokine signaling to suppress oncogenic behavior as a therapeutic strategy.

Expression of individual members of this negative regulatory cohort fluctuate during tumor onset and progression in a frequently tissue-specific manner, impacting both the duration and amplitude of TGF-β1 signaling. Disease outcome as a consequence of repressor deregulation appears to be determined, at least in part, by the specific role of TGF-β1 in particular cancer types. In cases where TGF-β1 hyperactivation promotes oncogenesis, loss of negative regulators (e.g., PPM1A, Klotho) is causatively linked to tumor progression and a poor prognosis [51,84]. In cancers where attenuation of TGF-β1 signaling accelerates tumor growth, upregulation of negative regulators such as SMAD7 could promote TGF-β1 receptor degradation and SMAD2/3 inactivation facilitating creation of an aggressive phenotype. An increase in SMAD7 gene copy number in colon cancer, for example, is associated with poor outcomes while deletion of SMAD7 confers a more favorable prognosis [66]. While the mechanism of SMAD7 deregulation in colon cancer is not clear, clarification of the underlying pathway(s) may inform development of novel strategies to repress SMAD7 levels as a therapeutic approach to disease management. In sharp contrast to the observations in colon cancer, increasing SMAD7 stability may have potential beneficial effects against progressive breast carcinoma since loss of OTUD1 expression destabilizes SMAD7 by ubiquitination promoting, thereby, TGF-β1-driven metastasis [64]. Such potential opposing outcomes of SMAD7 targeted therapy in two different cancer types underscores the emerging importance of individualized approaches to cancer treatment.

It is increasingly evident that loss or aberrant expression/function of certain TGF-β1 pathway repressors (e.g., PTEN) during cancer progression impact genetic programs driven by unexpected consequences on control of the TGF-β1-SMAD2/3/4 signaling network [38,47]. Prostate-specific PTEN loss, for example, not only activates SMAD2/3 but promotes increased protein accumulation of SMAD2/3 and SMAD4 as well which are critical for establishing prostate cell senescence and fibrotic changes in the tumor microenvironment [38]. SMAD4 silencing in the PTEN-deficient mouse by-passed the senescence barrier and promoted lethal metastatic prostate cancer with 100% penetrance [38] highlighting the need to clarify the pathophysiologic impact of TGF-β1 pathway hyperactivation in the context of PTEN deregulation.

Different mechanisms are responsible for the aberrant function of TGF-β1 pathway negative regulators in cancer progression. Klotho loss of expression is linked to Klotho promoter hypermethylation and subsequent epigenetic silencing. PPM1A and PTEN deficiencies appear to occur at the level of protein degradation while SMAD7 is destabilized by ubiquitination, e.g., [52]. Although the mechanistic basis underlying controls on such divergent pathways is yet to be fully understood, identification of critical players involved in these deregulatory steps (e.g., methyl transferases in the case of Klotho loss, components mediating protein degradation of PTEN or PPM1A, micro-RNAs that downregulate BMP7) is necessary in any strategy directed at restoring the expression of these TGF-β1 negative regulators as new avenues to modulate the oncogenic consequences of cytokine signaling.

Expression rescue of suppressed or lost TGF-β1 pathway signaling negative regulators as a therapeutic modality can be complicated particularly since TGF-β1 itself can orchestrate activation or suppression of these same network regulators generating, and thereby, pathological signal amplification. In this regard, TGF-β1 could downregulate PTEN and Ski/SnoN expression [44,45,101], leading to elevated and/or sustained TGF-β1 pathway activation. A potential vicious cycle of by-directional cross-talk between TGF-β1 and its repressors must be fully explored in a cancer-type-specific fashion to devise clinically useful strategies to target oncogenesis for improved patient outcomes.

## Abbreviations

AMPK	AMP-activated protein kinase
AP-1	Activator Protein 1
ATM	Ataxia-telangiectasia mutated
BAMBI	Bone morphogenetic protein and activin membrane-bound inhibitor
BMP	Bone morphogenetic protein
CBP	CREB-binding protein
DNMT	DNA methyltransferases
EGFR	Epidermal growth factor receptor
EMT	Epithelial-mesenchymal transition
FGF	Fibroblast growth factor
FGFR	Fibroblast growth factor receptor
HCC	Hepatocellular carcinoma
HCV	Human hepatitis C virus
HECT	E6-associated protein carboxyl terminus
ID	Inhibitor of DNA binding
IGF	Insulin-like growth factor
MH1	MAD homology 1
MMP	Matrix metalloprotease
OTUD1	OTU domain-containing protein 1
P/CAF	P300/CBP-associated factor
PDAC	Pancreatic ductal adenocarcinoma
PIP2	Phosphatidylinositol-4,5-triphosphate
PIP3	Phosphatidylinositol-3,4,5-triphosphate
PI3K	Phosphoinositide 3-kinase
PPM1A	Protein phosphatase magnesium/ manganese-dependent 1A
PTEN	Phosphatase and tensin homologue
R-SMAD	Receptor-regulated SMAD
ROS	Reactive oxygen species
Ski	Sloan-Kettering Institute proto-oncogene
SMAD	Mothers against decapentaplegic homolog
Smurf	SMAD ubiquitination regulatory factors
SnoN	Ski related novel gene
TAZ	Transcriptional coactivator with PDZ-binding motif
TEAD	TEA domain family
TGF-β	Transforming growth factor β
TGF-βR	Transforming growth factor receptor
USF	Upstream stimulatory factor
USP26	Ubiquitin-specific protease 26
UUO	Unilateral ureteral obstruction
YAP	Yes-associated protein
